# Asian citrus psyllid adults inoculate huanglongbing bacterium more efficiently than nymphs when this bacterium is acquired by early instar nymphs

**DOI:** 10.1038/s41598-020-75249-5

**Published:** 2020-10-26

**Authors:** El-Desouky Ammar, Justin George, Kasie Sturgeon, Lukasz L. Stelinski, Robert G. Shatters

**Affiliations:** 1 USDA-ARS, United States Horticultural Research Laboratory, Fort Pierce, FL 34945 USA; 2 Entomology and Nematology Department, Citrus Research and Education Center, University of Florida, 700 Experiment Station Rd., Lake Alfred, FL 33850 USA

**Keywords:** Microbiology, Plant sciences, Zoology

## Abstract

The Asian citrus psyllid (*Diaphorina citri*) transmits the bacterium ‘*Candidatus* Liberibacter asiaticus’ (CLas), which causes huanglongbing (citrus greening) disease, in a circulative-propagative manner. We compared CLas inoculation efficiency of *D. citri* nymphs and adults into healthy (uninfected) citron leaves when both vector stages were reared from eggs on infected plants. The proportion of CLas-positive leaves was 2.5% for nymphs and 36.3% for adults. CLas acquisition by early instar nymphs followed by dissections of adults and 4th instar nymphs revealed that CLas bacterium had moved into the head-thorax section (containing the salivary glands) in 26.7–30.0% of nymphs and 37–45% of adults. Mean Ct values in these sections were 31.6–32.9 and 26.8–27.0 for nymphs and adults, respectively. Therefore, CLas incidence and titer were higher in the head-thorax of adults than in nymphs. Our results suggest that following acquisition of CLas by early instar *D. citri* nymphs, emerging adults inoculate the bacteria into citrus more efficiently than nymphs because adults are afforded a longer latent period necessary for multiplication and/or translocation of CLas into the salivary glands of the vector. We propose that CLas uses *D. citri* nymphs mainly for pathogen acquisition and multiplication, and their adults mainly for pathogen inoculation and spread.

## Introduction

The Asian citrus psyllid, *Diaphorina citri* Kuwayama (Hemiptera: Liviidae), is the primary vector of the phloem-limited bacterium *Candidatus* Liberibacter asiaticus (CLas), the putative causal agent of huanglongbing (HLB, citrus greening) disease. HLB is currently the most destructive citrus disease in many parts of the world including Asia, USA, Brazil, and Central America^1–3^. CLas can infect all commercial citrus cultivars causing major reductions in fruit quality, yield and lifespan of infected trees^2^. HLB has spread quickly in Florida-USA since its first detection in 2005, and is now threatening citrus production in Texas, California and other parts of the USA^4^.

CLas is transmitted in a circulative-propagative manner by *D. citri*; it is acquired by psyllid nymphs or adults during feeding on the phloem of infected citrus plants and ingestion of CLas into the psyllid’s gut, followed by its translocation and probable multiplication into various organs including the salivary glands^5–9^. During feeding/salivation of the vector, CLas presumably moves from the salivary glands with salivary secretions into citrus phloem resulting in pathogen inoculation of susceptible plants^9,10^. CLas is also transmitted transovarially to the eggs and nymphs from infected mothers but only at a very low rate, ≤ 1% (2–6% in pooled samples of eggs and 3rd- 5th instar nymphs)^6^. *D. citri* nymphs acquire CLas from infected citrus plants much more efficiently than adults^5,6,8^ likely because nymphs feed (ingest) from the phloem more frequently and for longer durations than adults^11^, although other factors including differences in innate immunity between nymphs and adults may also influence successful acquisition^12–14^*.*

*Diaphorina citri* adults that acquired CLas from infected citrus plants as nymphs have higher titers of CLas and are more likely to transmit/inoculate this bacterium than those acquiring the bacterium during the adult stage^5,8^. However, most of the previous investigations on CLas transmission by *D. citri* have been conducted using mainly adults for inoculation tests, whether these adults had acquired the bacterium during the nymphal or adult stages^5,6,8,15^. This was probably done for several reasons: (1) *D. citri* nymphs are much more fragile than adults and nymphal duration is much shorter than the adult life span^16^, (2) *D. citri* nymphs feed/probe (i.e. insert their stylets into host tissue) nearly continuously^11^, and thus are more difficult to handle or transfer without causing injury to their stylets/mouthparts during transmission experiments, and (3) Nymphs cannot fly and typically remain on the plant from which they were hatched, thus contributing in only a minor way to CLas transmission to other plants. However, there are epidemiological, molecular and/or behavioral questions about CLas transmission by *D. citri* that could be answered from a more complete understanding of acquisition/transmission rate comparisons between nymphs and adults.

In this work, we tested the inocualtivity of 4th instar nymphs and 1–4 week old adults of *D. citri* when both stages of the vector were reared on CLas-infected citrus plants for several generations. Our experimental design insured that tested nymphs and adults had access to infected plants at the youngest possible age (early first instar nymphs) without the difficulty of having to handle/transfer the very delicate first instar nymphs. In these tests, we used the excised leaf assay method developed and used earlier by Ammar et al.^17^ and Raiol-Junior et al.^18^. Complementary electrical penetration graph (EPG) measurements indicated that feeding behavior of *D. citri* on excised citrus leaves was indistinguishable from that observed on intact leaves of whole plants^11^. Our results support the hypothesis that *D. citri* adults inoculate CLas more efficiently than nymphs following acquisition of CLas by early instar nymphs because: (1) the titer and/or incidence of CLas in the salivary glands of emergent adults are higher than in nymphs, and (2) adults are afforded a longer latent period necessary for multiplication and/or translocation of CLas into the salivary glands of the vector. We propose that CLas uses *D. citri* nymphs mainly for pathogen acquisition and multiplication, and their adults mainly for pathogen inoculation and disease spread.

## Results

### CLas-infected *D. citri* adults are more efficient than nymphs in inoculating CLas into citrus leaves

In Expt. 1, we compared the inoculativity of *D. citri* nymphs (4th instar) versus that of adult males or females (1–2 week old) that had been reared for several generations on CLas-infected citron plants. The proportion of CLas-infected (qPCR positive) leaves, following a 4-day inoculation period (with 5 insects/leaf), was 2.5% (1/40 leaves) for nymphs and 36.3% (29/80 leaves) for adults, and this difference was highly significant (*P* = 0.0001 in X^2^ test). This difference was consistent in three out of four tests of Expt. 1 (Table 1). No significant difference was found between adult males and females with regard to the proportion of CLas positive leaves (32.5 and 40.0% for males and females respectively; *P* = 0.4839). The Ct value of CLas positive leaves was 25.5 for a single leaf inoculated by nymphs, and 26.6 ± 0.55 in leaves inoculated by adults.

**Table 1:** Percentage of CLas-infected (qPCR-positive) excised citron leaves that were inoculated by *D. citri* nymphs or adults for 4 days (5 insects/leaf, 10 leaves/treatment/test), then assayed by qPCR one week later (Expt. 1)*.

Test No	Leaves inoculated by			
	Nymphs	Adult males	Adult females	Adults (males + females)
1	0	40	40	40
2	0	60	90	75
3	0	20	20	20
4	10	10	10	10
Overall	2.5	32.5	40	36.3

*All inoculating nymphs and adults were reared on CLas-infected citron plants for several generations prior to the test.

In this experiment, the proportion of CLas-infected (qPCR positive) psyllids was 79.5% for nymphs (n = 200) and 91.4% for adults (n = 394) (*P* = 0.01). However, there was no significant difference in infection rate between male and female adults (Supplementary Table S1).

In summary, the results of Expt. 1 indicated that young (1–2 week old) *D. citri* adults reared on infected citrus for several generations exhibited a higher infection rate, and were more efficient in inoculating CLas into citrus leaves, compared to 4th instar nymphs reared similarly. Furthermore, there were no differences in inoculation efficiency or infection rate between adult males and females reared on infected citrus.

### CLas incidence and titer in the head-thorax and abdomen sections are higher in adults than in nymphs of CLas-infected *D. citri*

The salivary glands of *D. citri* are found in the head and first two thoracic segments, and we postulated that entry and/or multiplication/accumulation of CLas in the salivary glands are essential for CLas transmission and inoculation to occur. To test this hypothesis, we compared the incidence and titer (as measured by normalized Ct values) of CLas in the head-thorax and abdomen sections of nymphs and adults separately in Expts. 2 and 3.

In Expt. 2, which was comprised of two tests (with 30 nymphs and 40 adults/test), the proportion of CLas-infected (qPCR positive) anterior (head-thorax) sections in the adults (45%, n = 80) was significantly higher than that in nymphs (26.7%, n = 60, *P* = 0.040). Also, a significantly higher proportion of infected abdomens was found in adults (92.5%) than in nymphs (65%, *P* = 0.0001). Both nymphs and adults had a significantly higher proportion of infected abdomens than infected head-thorax sections (*P* = 0.0001 and 0.0002 for adults and nymphs, respectively).

In this experiment, the CLas titer found in infected adults was significantly higher (= lower Ct values) than in nymphs in both the anterior (head-thorax) and posterior (abdomen) sections (Table 2). ANOVA on Ct values indicated that the effect of life stage (nymphs vs. adults) on CLas titer was highly significant (*P* = 0.0000), but the effect of body section and the interaction between these two factors were not significant (*P* = 0.992 and 0.992, respectively).

**Table 2:** Normalized Ct values of CLas in the anterior (head-thorax) and posterior (abdomen) sections of *D. citri* nymphs and adults that were reared on CLas-infected citron plants (Expts. 2 and 3).

Expt. no	Body part	Nymphs			Adults			*P* (T test)
		N	Mean	SE	N	Mean	SE	
2*	Head-thorax	16	32.85	1.1	36	26.95	0.71	0.0001
	Abdomen	39	32.01	0.77	74	27.81	0.56	0.0001
3**	Head-thorax	9	31.63	0.85	22	26.80	0.55	0.0001
	Abdomen	17	32.35	0.80	52	29.37	0.46	0.0020

*Pooled data from two tests; also results from males and females were pooled since gender had no significant effect on Ct values.

**Results from younger and older adults in Expt. 3 were pooled since their Ct values did not differ significantly (Table 3).

**Table 3:** Normalized Ct values of CLas in the head-thorax and abdomen sections of younger and older *D. citri* adults that were reared for several generations on CLas-infected citron (Expt. 3).

Body part	1–2 week adults			3–4 week adults			*P* (T test)
	N	Mean	SE	N	Mean	SE	
Head-thorax	11	26.76	0.89	11	26.84	0.89	0.9477
Abdomen	28	29.49	0.67	24	29.23	0.72	0.7931

In Expt. 3, we compared 4th instar nymphs vs. 1–2 week and 3–4 week old adults to determine if adult age may affect the infection rate or CLas titer. No differences were found between younger and older adults in the proportion infected or in CLas titer for both the abdomen and head-thorax sections (Table 3). However, the proportion of CLas-infected abdomens was significantly higher in adults (86.7%) than in nymphs (56.7%, *P* = 0.006). With regard to the head-thorax section, the proportion infected was higher in adults (36.7%) than in nymphs (30%) but this difference was not significant (*P* = 0.532). Additionally, ANOVA on the data summarized in Table 2 (Expt. 3) indicated that the effect of life stage on CLas titer in both body sections was highly significant (*P* = 0.0000), the effect of body section was significant (*P* = 0.0302), but the interaction between these two factors was not significant (*P* = 0.221).

In summary, the results of Expts. 2 and 3 indicated that *D. citri* adults reared for several generations on CLas-infected citron plants exhibited higher incidence and titer of CLas than did nymphs reared similarly, when measured either in the head-thorax (which includes the salivary glands) or abdomen sections. Additionally, there were no differences in infection rate or CLas titer in these sections between younger (1–2 week) and older (3–4 week) psyllid adults.

### Feeding behavior of *D. citri* nymphs and adults on excised citron leaves as measured by EPG recordings

*Diaphorina citri* nymphs have been reported to ingest from, and salivate into, the phloem of healthy or CLas-infected citron plants more frequently and for longer durations than adults^11^. However, in that study only healthy psyllids (previously unexposed to CLas) were tested on intact leaves of citron plants. In the present work, we investigated the feeding/probing activities of CLas-infected *D. citri* nymphs and adults using the same EPG equipment, recording duration (42 h) and methodology as that in George et al.^11^ except for using excised (rather than intact) healthy citron leaves.

**Figure 1:**
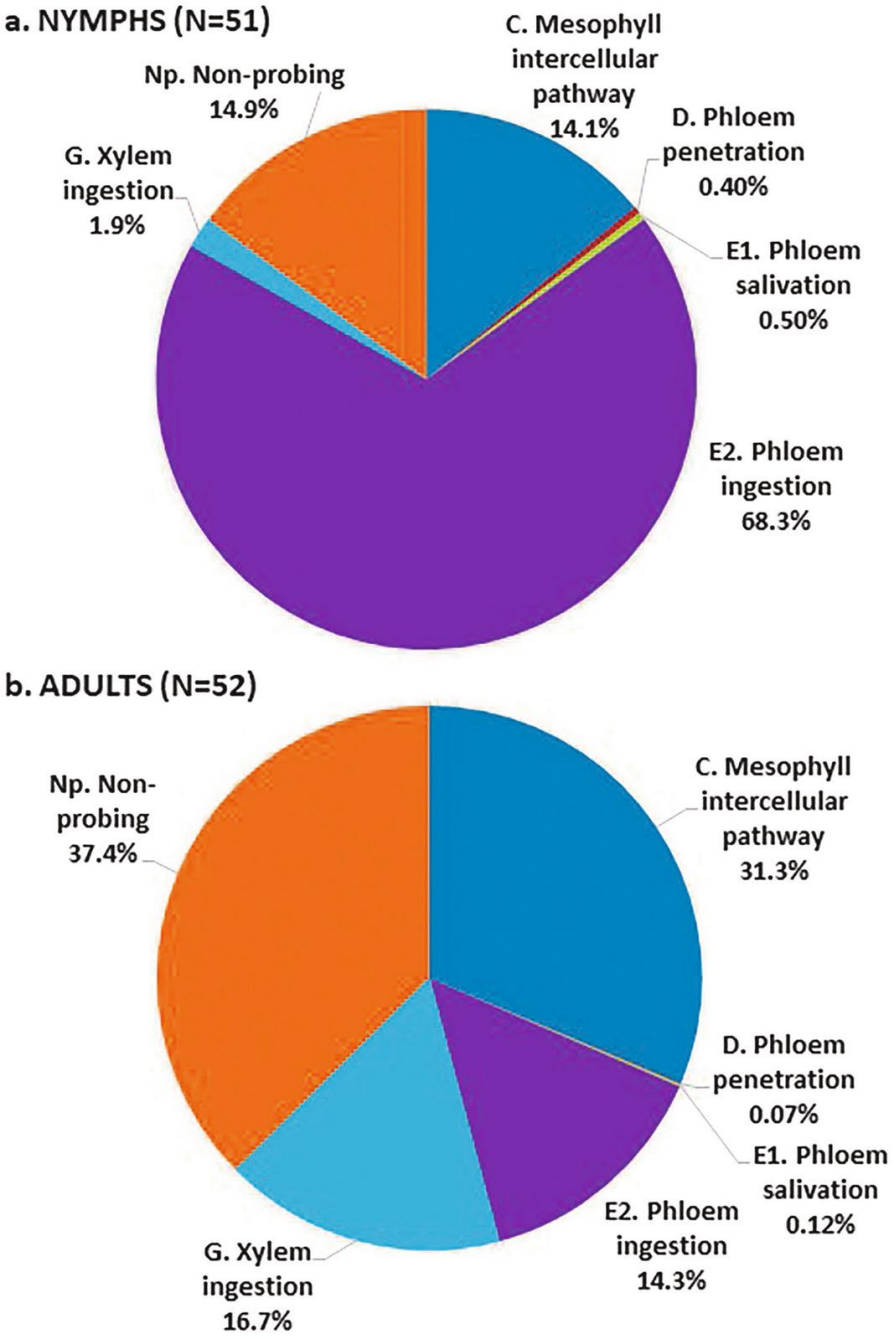
Graphic presentation of various feeding/probing activities (waveforms) of CLas-exposed *D. citri* nymphs (**a**) and adults (**b**) on excised healthy citron leaves. EPG recordings were performed for 42 h. The total duration for each activity is indicated by percentage in the pie charts. The data represent the average of 51 nymphs and 52 adults.

Our EPG results (Table 4, Fig. 1) were congruent with those obtained previously by George et al. ^11^. In short, the duration and frequency of phloem salivation and ingestion phases (E1 and E2 waveforms, respectively) recorded from CLas-exposed *D. citri* nymphs (4th–5th instar, n = 51) were significantly greater than those recorded from CLas-exposed adults (1–2 week old, n = 52). The percentages of CLas-infected (qPCR positive) nymphs and adults used in this EPG study were 87.5 and 100% respectively, with mean Ct values of 35.97 ± 0.48 and 29.31 ± 0.52 for nymphs and adults, respectively. The difference in the proportion of infected nymphs and adults was not significant (*P* = 0.07), but the difference in mean Ct values was highly significant (*P* = 0.001), indicating higher CLas titer in adults than in nymphs as we reported above in Expts. 1–3.

**Table 4:** Electrical penetration graph (EPG) recordings of feeding/probing activities of CLas-exposed *D. citri* nymphs (4th–5th instar, n = 51) and adults (n = 52) on excised, healthy, young citron leaves over 42 h: no. of bouts (count), mean duration (minutes) and total duration (count × mean duration) for each waveform (data analyzed by non-parametric Kruskal–Wallis test).

Waveform	Variable	Nymph	Adult	Chi square-value	P-value
C. Mesophyll intercellular pathway	Count	30.7 ± 2.9	40.0 ± 3.6	3.66	0.05
	Duration (min)	16.6 ± 2.0	38.4 ± 8.3	7.82	**0.005**
	Total duration (min)	374.0 ± 32.4	841.5 ± 72.4	32.03	** < 0.0001**
D. Phloem penetration	Count	10.0 ± 0.8	2.2 ± 0.4	53.02	** < 0.0001**
	Duration (min)	1.2 ± 0.2	0.5 ± 0.1	19.12	** < 0.0001**
	Total duration (min)	10.7 ± 1.2	1.8 ± 0.4	56.7	** < 0.0001**
E1. Phloem salivation	Count	10.0 ± 0.8	2.2 ± 0.4	52.5	** < 0.0001**
	Duration (min)	1.4 ± 0.1	1.2 ± 0.3	9.75	**0.002**
	Total duration (min)	13.0 ± 1.3	3.2 ± 0.6	42.39	** < 0.0001**
E2. Phloem ingestion	Count	7.3 ± 0.6	1.9 ± 0.4	45.3	** < 0.0001**
	Duration (min)	410.0 ± 55.3	173.0 ± 40.7	25.3	** < 0.0001**
	Total duration (min)	1823.2 ± 93.8	385.2 ± 44.1	59.58	** < 0.0001**
G. Xylem ingestion	Count	1.6 ± 0.6	9.3 ± 1.3	52.85	** < 0.0001**
	Duration (min)	23.0 ± 4.6	68.1 ± 10.8	29.87	** < 0.0001**
	Total duration (min)	49.4 ± 71.4	449.7 ± 56.5	61.92	** < 0.0001**
Np. Non-probing	Count	18.7 ± 2.1	26.4 ± 3.1	3.05	0.08
	Duration (min)	26.2 ± 5.1	59.3 ± 9.8	15.82	** < 0.0001**
	Total duration (min)	397.8 ± 62.7	1000.4 ± 88.8	23.50	** < 0.0001**

During the 42 h recording period, nymphs performed significantly more phloem ingestion bouts (7 ± 1) than did adults (2 ± 1). On the other hand, adults engaged in more bouts of xylem ingestion (9 ± 1) than nymphs (2 ± 1). Additionally, insect stage had a significant effect on the mean and total duration of all waveforms (Table 4, Fig. 1). The duration of individual bouts of waveforms D, E1 and E2 (all related to phloem feeding activities) were significantly longer for nymphs than adults. Total duration of phloem ingestion (E2) was significantly longer in nymphs (1823 ± 94 min) than in adults (385 ± 44 min), as was the total duration of phloem salivation for nymphs (13.0 ± 1.3 min.) compared to adults (3.2 ± 0.6 min.). Nymphs were engaged in phloem ingestion 5 times longer, on average, than were adults; but the adults spent 9 times longer engaged in xylem ingestion compared with nymphs. During the 42-h recordings, nymphs spent 68% of their time (30.4 h) engaged in phloem ingestion, whereas adults spent only 14% of their time (6.4 h) in phloem ingestion. On the other hand, adults spent 17% of the total feeding time engaged in xylem ingestion, whereas nymphs spent only 2% of their time in xylem feeding activities (Fig. 1).

In summary, our EPG results indicated that feeding behavior of CLas-infected *D. citri* nymphs and adults on excised leaves was indistinguishable from that described previously for unexposed *D. citri* on intact citrus leaves^11^. These results indicate that differences in inoculation rate between CLas infected *D. citri* nymphs and adults, reported above, are not associated with differences in feeding behavior on excised versus intact citrus leaves.

## Discussion

The circulative-propagative mechanism of transmission that occurs among hemipteran-borne plant pathogen vectors has been hypothesized to occur in the CLas/HLB pathosystem^7,10^. This mechanism is comprised of several phases that include: (a) ingestion of the pathogen and its movement into the vector’s gut during phloem feeding on an infected host plant, (b) translocation of the pathogen from the gut into the hemolymph and various tissues including the salivary glands with probable pathogen multiplication in these tissues, and (c) inoculation of the pathogen by injection within salivary secretions during subsequent feeding on a susceptible host^7,10,19^. However, prior to inoculation, there is a ‘latent period’, during which the pathogen translocates into, and presumably multiplies in, the salivary glands. The minimum latent period for CLas in *D. citri* that acquired CLas as nymphs ranged between 7 and 10 days, with a median of 16.8 days^8,20^. The total nymphal duration of *D citri* at 25 °C averages 12.8 days^16^. This is much shorter than the median latent period reported for CLas, and hence it is expected that most of CLas inoculations occur at the adult stage, rather than during the preceding nymphal stage of the vector, as our results here suggest.

In the present work, we used *D. citri* that were reared for several generations on CLas-infected citrus plants. Since transovarial transmission of CLas in *D. citri* may occur at a very low rate approaching 1%^6^, it is likely that the great majority of *D. citri* nymphs and adults used here had acquired CLas by feeding on infected plants as early instar nymphs. Our results indicate that CLas-infected adults were more efficient than nymphs in subsequently inoculating this pathogen into citrus leaves. Congruently, these adults were characterized by greater CLas incidence and higher CLas titer (lower Ct values), than counterpart nymphs, in the head-thorax body section that included the salivary glands. In *D. citri* head and first 2 thoracic segments, other than the salivary glands, there are mainly neural tissues (the brain and compound ganglionic mass) which are rarely infected with CLas^9,23,26^. Although CLas incidence and titer was also higher in the abdomen section of adults, compared to nymphs, this difference was not consequential to the inoculation process, which requires presence of CLas in the salivary glands according to the circulative and circulative-propagative model^7,10,19^. Additionally, it has been reported that overall CLas titer (in the whole psyllid) is positively correlated with higher inoculation rates on excised leaves or intact citrus plants^15,17^. Similarly, transmission experiments using field-collected *D. citri* in Japan suggested that CLas-transmitting insects tend to exhibit higher bacterial densities than do non-transmitting insects, and that a threshold level of CLas density in the psyllid (ca.10^6^ bacterial cells per insect) is required for successful transmission to occur^21^. In these investigations, CLas titer in the salivary glands was unknown; however, Wu et al.^22^ reported that when CLas was acquired by *D. citri* nymphs the proportion of infected hemolymph and salivary glands, as well as their CLas titer, increased significantly 12–18 days following a 3-day acquisition access period. These results suggest that CLas multiplies and/or accumulates in the vector’s hemolymph and salivary glands during the latent period.

The higher CLas incidence (infection rates) and titers measured from the head-thorax sections of adults compared to nymphs is consistent with our hypothesis that greater transmission efficiency observed in adults than nymphs is related to the latent period during which the pathogen must translocate to, and/or multiply in, the salivary glands before inoculation can occur. This latent period may be incomplete during nymphal development, which requires less time to complete than the median reported CLas latent period in this vector^16,20^. Our results also indicated that the proportion of infected head-thorax sections was considerably lower than that of infected abdomens in both nymphs and adults. This is consistent with previous findings indicating that the infection rates of salivary glands from field-collected or laboratory-infected *D. citri* adults are lower than those of infected guts or other tissues^23,24^. Similarly, Cooper et al.^25^ found that fewer salivary glands of the potato psyllid were infected with *Candidatus* Liberibacter solanacearum (CLso) compared with the alimentary canals from those same individuals. These results suggest the existence of transmission barriers that may impede CLas and CLso from exiting the gut and/or entering salivary glands in some vector psyllids of these phytopathogens^10,26^. These barriers are probably more permissive in *D. citri* nymphs than in adults, which may also explain that CLas multiplies faster in nymphs than in adults^5,8,10^.

In our inoculativity tests, we used the excised leaf assay method developed and used earlier by Ammar et al.^17^ and Raiol-Junior et al.^18^, because it saves considerable time, space and material compared to using whole plants (qPCR results can be obtained in 2 weeks rather than ≥ 6 months post-inoculation). Here, this method was also much more suitable for testing nymphs because they were confined into a much smaller space (in a 50 mL tube) which reduced their chance of getting lost or dropping off the plant during the test. Inoculation rates (% CLas-positive leaves or plants) using these two methods were comparable in two previous studies^17,18^. Furthermore, the EPG recordings conducted here indicated no differences in feeding behavior of *D. citri* on excised citrus leaves, compared to those made previously using intact leaves on whole plants^11^. In both studies, the occurrence (frequency) and duration of both E1 and E2 waveforms, representing phloem salivation and ingestion phases respectively, were much greater in nymphs than in adults. Previous EPG studies indicated that CLas acquisition occurs during phloem ingestion and that CLas inoculation occurs during phloem salivation phases^27,28^.

In our experiments, there was no differences in infection rates or titers measured from 1 to 2 week versus 3 to 4 week old adults. These results are perhaps unsurprising, since multiplication of CLas in *D. citri* adults is known to occur much more slowly than in nymphs^5,8^. Also, there were no gender related differences in the proportion of insects that were CLas-infected, inoculative, or in their CLas titer. Similarly, no gender differences were reported from laboratory reared, CLas-infected *D citri* adults^15,22^, but the proportion of infected females was generally higher than that of males in field-collected samples from Florida^29^. Since *D. citri* females live much longer than males^30^ this might affect their rate of infectivity under field conditions, which may bias the proportion of infected psyllids slightly toward females.

Epidemiologically, it is interesting to note that although *D. citri* nymphs are much more efficient than adults in CLas acquisition from infected plants^5,6,8,11,22^, adults appear to be more important in CLas inoculation and disease spread because of three factors: (a) their greater mobility compared to nymphs^3^, (b) longer occurrence within the lifecycle (adult life span vs. nymphal duration)^16^, and (c) greater efficiency of CLas inoculation by adults compared to nymphs as we reported here. Lee et al.^31^ proposed the model of ‘flush transmission’ of CLas between infected *D. citri* adults and their young nymphs via the host plant when these nymphs feed on newly flushing leaves that became recently infected by their parents. However, they did not specify whether CLas inoculation during the second generation (that acquired CLas from the flush) occurs during the nymphal or adult stages. Based on our results, it seems reasonable to assume that most of these 2nd generation psyllids would inoculate CLas mainly during their adult life. This also increases the significance of adults for spreading CLas infection in the field. Thus, in an evolutionary sense, CLas seems to have taken maximum advantage of both stages of its vector *D. citri*, using the nymphal stage mainly for acquisition and pathogen multiplication in the vector^5,8,22^, and the adult stage mainly for pathogen inoculation and short or long-distance disease spread. This apparent specialization of the two developmental stages of *D. citri* may have been aided by differences in innate immunity between nymphs and adults, as suggested by quantitative proteomic and microscopic studies of uninfected and CLas-infected *D. citri*^12–14^.

Although the rates of CLas inoculation per insect, especially in Florida, is fairly low^6,8,10,17^ this is more than compensated for by the great reproductive potential and short generation time of *D. citri*^3,16^ in addition to the beneficial effects of this bacterium on its psyllid vector^32^. Our work helps to further elucidate the great degree of adaptation between CLas and its primary vector and the different roles played by nymphs and adults of *D. citri* in the transmission cycle of CLas. These differences should be taken into consideration when devising methods for combating HLB, the most serious citrus disease worldwide.

## Methods

### Psyllids and plants used

*Diaphorina citri* nymphs and adults originated from a healthy (uninfected, non CLas-exposed) laboratory colony maintained for several years in the ARS-USDA Laboratory in Fort Pierce, FL. on healthy (uninfected) orange jasmine [*Murraya paniculata* (L.) Jack] trees, and more recently on healthy (uninfected) citrus trees (*Citrus macrophylla* Wester) in the greenhouse. Individual adults from the colony were sampled and qPCR-assayed every three months to ensure that the colony remained CLas-free. CLas-exposed (potentially infected) *D. citri* nymphs and adults were obtained by rearing uninfected *D. citri* from eggs on young CLas-infected citron plants (*Citrus medica*) that tested positive for CLas by qPCR. Routine qPCR was conducted on healthy and infected colony insects and citrus trees using Li primers^33^. Healthy (uninfected) citron plants were grown from seeds under insect-proof greenhouse conditions, and their excised leaves were used for testing CLas inoculation by *D. citri* nymphs and adults as described below.

### CLas-infected *D. citri* nymphs and adults

*Diaphorina citri* has five nymphal instars that can be distinguished morphologically and by size^3^. Fourth instar nymphs and 1–2 or 3–4 week old adults were selected from known CLas-positive *D. citri* colonies, with high CLas titers (= lower Ct values, typically Ct values below 28 for single adult samples and an infection rate typically greater than 70%) as evidenced by previous qPCR tests. Fourth instar nymphs were chosen for our experiments because they are hardier than earlier instars, allowing handling with less chance of damage, while allowing at least 4 days of testing prior to molting into adults^16^. These nymphs were manipulated with a moistened fine paint brush under a stereomicroscope. *D. citri* nymphs are known to insert their stylets into citrus leaves and feed/probe continuously for several hours^11^. Thus, in order to avoid injuring their delicate mouthparts, they were “tickled” gently with the paint brush until they stopped probing and began to move. They were then gently picked up with the paint brush and placed on the underside of excised healthy young citron leaves, and then caged within modified conical tubes as described below.

One to 2 or 3–4 week old *D. citri* adults were collected from the same CLas-positive colony as nymphs for use in the same experiment. Adults were collected with an aspirator and placed at 4° for 10–20 min to briefly immobilize them. They were then examined under a stereomicroscope to determine their sex. In some tests, males and females were separated and placed in tubes with 5 insects/leaf/tube. However, earlier tests showed no significant differences between adult males and females with regard to CLas infectivity or inoculativity (see “Results”); thus in later tests mixed gender adults were used.

### Experiment 1: testing inoculativity of D. citri nymphs and adults after rearing on CLas-infected plants

Healthy, young citron leaves were used for testing the inoculativity of *D. citri* nymphs and adults that had been reared for several generations on CLas-infected citron plants, using a slightly modified excised leaf method developed and used earlier by Ammar et al.^17^ and Raiol-Junior et al.^18^. Fully expanded young leaves (5–7 cm long) were selected from healthy (uninfected) citron plants. They were excised by a razor blade with their petioles intact. Leaf petioles were then placed under water and cut again with a razor blade at 45° angle. Leaves were immediately placed in a modified conical base of 50 mL conical tubes with 3 mL of sterile water (Supplementary Fig. S1). The base/bottom part of the tube had been cut earlier to facilitate adding more water when necessary without disturbing the feeding psyllids in the tube. Parafilm membrane was used to maintain contact between the base and remaining part of the tube except when water was added. Five *D. citri* nymphs (4th instar), or adult males or females (1–2 week old) were placed on each leaf, after which tubes were capped with a screen screw top^17^. The nymphs or adults placed on an excised leaf were allowed to move freely and to select their preferred feeding sites on that leaf during the 4 days of inoculation. These tube-caged leaves were kept on a lab bench for 4 days under a grow light (24 °C, 14 h of light) before the insects were removed, frozen, then tested later individually for CLas by qPCR. The 4-day inoculation period was chosen so that most of the 4th instar nymphs tested did not molt into adults prior to the conclusion of the test, and 5 insects/leaf was chosen because it yields much higher inoculation rates than using a single insect/leaf^17^. At the conclusion of each test, leaves in the conical tubes were placed into an illuminated incubator at 25 °C for 7 days, which is known to increase CLas titer in these leaves^17^. Thereafter, the leaves were washed thoroughly before being tested for CLas with qPCR essentially as described earlier^8,17^. The main difference was that total nucleic acid extractions containing both DNA and RNA were analyzed using q-RT-PCR (quantitative-reverse transcriptase- PCR) instead of just qPCR. We have found that detection of both DNA and RNA in q-RT-PCR reactions is a more sensitive method of detecting CLas transmission to plant leaves. This analysis was performed using the same cycling parameters as previously described with the exception of using the GoTaq 1-Step RT-qPCR System (Promega, Inc., Madison, WI) and their one-step pre-incubation cDNA synthesis recommendation.

In Expt. 1, the inoculativity test on excised leaves (5 insects/leaf, 10 leaves/ treatment/test) was repeated four times. The proportion of insects that survived at the end of the 4-day tests was 90% for nymphs (n = 200) and 94.8% for adults (n = 400). Also, at the end of the 4-day test, 26.1% of the nymphs used emerged as adults.

### Experiments 2 and 3: Testing the anterior (head-thorax) vs. posterior (abdomen) parts of nymphs and adults by qPCR

The two salivary glands of *D. citri* are located in the head and first two thoracic segments^23,24,26^. Thus, in order to test the hypothesis that differences in the occurrence and/or titer of CLas in the salivary glands may contribute to differences in CLas inoculativity between *D. citri* nymphs and adults, we performed qPCR tests on the anterior (head-thorax) and posterior (abdomen) sections of each insect separately. Based on preliminary trials, we estimated that dissecting and handling the tiny and delicate salivary glands of a large number of psyllids, especially nymphs, would be challenging logistically, and some of these tiny salivary glands could be damaged or lost during dissection or processing^24^. Thus, dissecting the thorax-head part was determined to be a suitable compromise, especially that the main tissues in *D. citri* head and first 2 thoracic segments, other than the salivary glands, are neural tissues (the brain and compound ganglionic mass) which are rarely infected with CLas^9,23,26^.

In Expt. 2 we tested 4th instar nymphs vs. 1–2 week old adults (two tests, with 30 nymphs and 40 adults/test), while in Expt. 3 we tested 4th instar nymphs vs. 1–2 or 3–4 week old adults (n = 30 for each category). Fourth instar nymphs and 1–4 week old adults were selected from the same CLas positive colony as described above. Under a stereomicroscope, each insect was carefully cut between the second and third thoracic segments with a clean sharp razor blade (a new blade was used for each insect). For simplicity, we refer to these anterior and posterior sections as ‘head-thorax’ and ‘abdomen’, respectively, although the posterior section included the 3rd thoracic segment in addition to the full abdomen. Each section was placed in a separate 1.5-mL tube, frozen on liquid nitrogen and stored at − 80 °C until extractions were conducted. For DNA extraction, samples were ground with a small tube pestle in liquid nitrogen, and then homogenized further with pestles in 150 µL TE buffer (10 mM Tris–HCL, 1 mM EDTA pH 8.0). Then 150 µL of phenol (equilibrated with 10 mM Tris–HCL pH 8.0) was added and the samples were vortexed. The aqueous phase was removed and DNA was precipitated by addition of 0.5 volumes of 5 M ammonium acetate and 2 volumes of 95% ethanol followed by centrifugation for 15 min at 21,000×*g*. The DNA pellet was washed with 300 uL of ice-cold 70% ethanol, allowed to air dry for 10 min and then resuspended in 30 µL of sterile deionized water and stored at − 80 °C until used. The qPCR parameters for CLas detection were as described previously^8^. Normalization of CLas 16S rRNA Ct values to psyllid genome equivalents was performed by simultaneous qPCR detection of the CLas 16S rRNA gene and the *D. citri* Ribosomal S20 (RPS20) psyllid gene (Genbank accession #DQ673424) used as an internal reference: Dci-S20-L:5′- GCCCAAGGGCCCAATCA -3′, and Dci-S20-R:5′- GGAGTCTTACGGGTGGTTATTCTG-3′. Normalization was performed by subtracting the Ct value for the individual with the lowest Dci-S20 Ct value from all the individual Dci-S20 Ct values and then the resulting number from each individual was subtracted from that individual’s CLas Ct value. For statistical analysis on the results of Expts.1–3, X^2^ tests were conducted on the proportion of CLas-infected (qPCR positive) leaves or psyllids, while T tests and/or ANOVA were conducted on the Ct values of the individual psyllids tested by qPCR, using the stat. analysis program JMP (v. 10, SAS Inc., 541 Cary, NC). It has been established earlier that Ct values are negatively correlated with CLas titer in the psyllids and leaves tested ^8^.

### Experiment 4: Feeding behavior of CLas-infected *D. citri* nymphs and adults on excised citron leaves measured by electrical penetration graph (EPG) recordings

*Diaphorina citri* nymphs (4th instar) and adults (1–2 week old) used in the EPG study were selected from psyllid colonies with high CLas titers (low Ct values). Previous EPG recordings indicated no differences in EPG waveforms produced by adult males or females of *D. citri*^11,34^. Therefore, the sex of adults used in this study was not determined. EPG recordings were performed on *D. citri* nymphs or adults feeding on excised leaves from healthy (uninfected) citron plants grown from seeds. Young leaves (soft, fully expanded, ca. 5 cm long and 3 cm wide) were selected. Leaves with their petioles were excised from the plant; the leaf surface was cleaned and air dried, and the leaf petiole was introduced into a 4 mL glass vial filled with water.

EPG recordings were obtained using a DC-monitor, GIGA-8 model, EPG-Systems, Wageningen, and the Netherlands, adjusted to 50 × gain. The analog signal was digitized through a DI-710 board and displayed using Windaq Lite ver. 2.40 software (Dataq Instruments Inc. Akron, OH, USA) on a Dell desktop computer. The EPG monitoring system was housed in a grounded Faraday cage in an environmentally controlled room under continuous lighted conditions. Temperature was set to 26 °C with 60–65% RH. Psyllid nymphs or adults were collected 4 h prior to the start of the experiment each day and were starved for this period inside glass vials. Adult psyllids were then placed in a freezer (− 4 °C) for 45–60 s to immobilize them, then held by a plastic pipette tip connected to a gentle vacuum supply under a dissecting microscope. Each psyllid nymph or adult was attached to a 25 μm-diam. gold wire (Sigmund Cohn Corp., Mt. Vernon, New York) by a droplet of silver conducting paint (Ladd Research Industries, Burlington, VT) applied to the insect’s pronotum (Supplementary Fig. S2). The gold wire lead was attached to a copper electrode (3 cm × 1 mm diameter) connected to the EPG probe. To complete the electrical circuit, a reference copper electrode (10 cm × 2 mm) was inserted into the water in the vial containing the leaf petiole. Psyllid nymphs and adults were restricted to the abaxial (lower) surface of the leaf, the preferred feeding site for nymphs^3,11^. The feeding behavior of individual *D. citri* (51 nymphs and 52 adults) was monitored for a continuous period of 42 h. This is the same recording duration we have used in our previous CLas acquisition study on *D. citri* nymphs and adults^11^ and is much longer than most EPG studies for *D. citri* reported previously.

Following the 42 h recordings, all tested insects were kept individually in vials with 90% ethanol and stored in a refrigerator until DNA extraction and qPCR testing. Characterization of EPG waveforms was accomplished by visually identifying and annotating waveforms based on comparison with prior EPG and histological studies on *D. citri*^11,25,34^. Windows Dataq waveform browser (Dataq Instruments Inc., Akron, OH) was used to annotate waveforms. The number and duration of waveform bouts were tabulated in an electronic spreadsheet. The waveforms were visually inspected for frequency patterns and annotated as non-probing (Np), mesophyll intercellular pathway (C), phloem penetration (D), phloem salivation (E1), phloem ingestion (E2) or xylem ingestion (G) phases. Statistical analysis was performed using JMP (v. 10, SAS Inc., Cary, NC). Data analyzed by non-parametric test, Kruskal–Wallis test (n = 51 for nymphs, n = 52 for adults, α = 0.05) without differentiating insect sex (v. 10, SAS Inc., Cary, NC, USA).

### Ethics

All experimental protocols were approved by the Shatters Lab., US Horticultural Research Laboratory, Fort Pierce, Florida, USA. All methods were carried out in accordance with relevant guidelines and regulations approved by Agric. Res. Service, USDA.

## Supplementary information

Supplementary Information.

## Data Availability

Experimental data generated during the current study are available from the corresponding author upon request.
