# Supplementary material for: Asian citrus psyllid adults inoculate huanglongbing bacterium more efficiently than nymphs when this bacterium is acquired by early instar nymphs
Source: Sci Rep. 2020 Oct 26;10:18244. doi: 10.1038/s41598-020-75249-5 (PMC7589476; doi:10.1038/s41598-020-75249-5)

Supplementary Material

Title: Asian citrus psyllid adults inoculate huanglongbing bacterium more efficiently than nymphs when this bacterium is acquired by early instar nymphs

Authors: El-Desouky Ammar[1*], Justin George[1,2], Kasie Sturgeon[1,2], Lukasz L. Stelinski[2] and Robert G. Shatters[1]

[1]USDA-ARS, United States Horticultural Research Laboratory, Fort Pierce, FL 34945, USA

[2]University of Florida, Entomology and Nematology Department, Citrus Research and Education Center, 700 Experiment Station Rd., Lake Alfred, FL 33850, USA

*Corresponding author (desoukyammar@gmail.com)

Supplementary Table S1. Proportion and percentage of CLas-infected (qPCR positive) nymphs and adults, reared previously on infected citron, following 4 days of feeding on excised healthy citron leaves (5 insects/leaf, 10 leaves/treat./test, Expt.1)

Test No.	Nymphs		Males		Females		Adults (M + F)	
	Positive /total	% positive	Positive/ total	% positive	Positive/ total	% positive	Positive/ total	% positive
1	42/50	84.0	46/50	92.0	43/50	86.0	89/100	89.0
2	31/50	62.0	47/47	100.0	47/47	100.0	94/94	100.0
3	50/50	100.0	37/50	74.0	42/50	84.0	79/100	79.0
4	36/50	72.0	48/50	96.0	50/50	100.0	98/100	98.0
Overall	159/200	79.5	178/197	90.4	182/197	92.4	360/394	91.4

Supplementary Figure S1

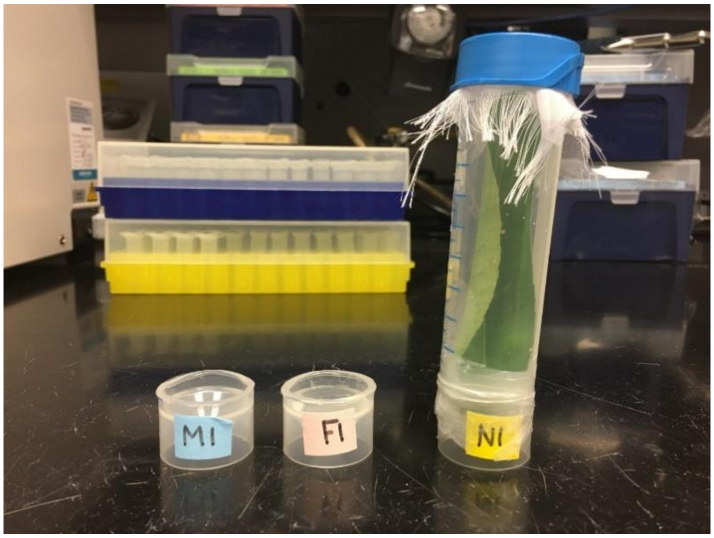

Supplementary Figure S1. Modified conical 50 mL tubes used for testing the inoculativity of *D. citri* nymphs and adults on excised citron leaves. Two modified conical bases are on the left, and a full leaf setup in a modified conical tube on the right. M, males; F, females, N, nymphs.

Supplementary Figure S2

Supplementary Figure S2
D. citri 4th instar nymph (a) and 1-2 wk old adult (b) feeding on citrus leaves; each insect is tethered with a gold wire (arrow) connected to an electrical penetration graph (EPG) recorder to monitor their feeding/probing activities for 42 h.

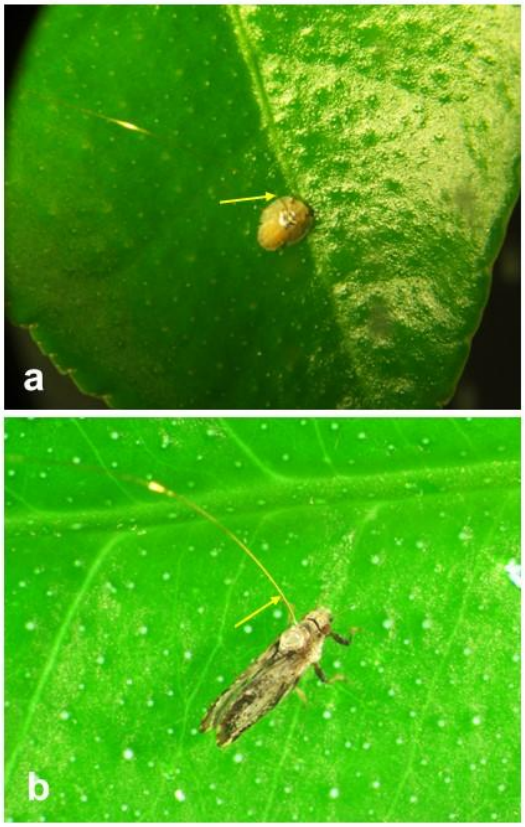

Supplement: Supplementary file 1 — Supplementary Information. [file 41598_2020_75249_MOESM1_ESM.pdf]
